# A large dataset of protein dynamics in the mammalian heart proteome

**DOI:** 10.1038/sdata.2016.15

**Published:** 2016-03-15

**Authors:** Edward Lau, Quan Cao, Dominic C.M. Ng, Brian J. Bleakley, T. Umut Dincer, Brian M. Bot, Ding Wang, David A. Liem, Maggie P.Y. Lam, Junbo Ge, Peipei Ping

**Affiliations:** 1 The NIH Big Data to Knowledge (BD2K) Center of Excellence in Biomedical Computing at UCLA, Los Angeles, California 90095, USA; 2 Department of Physiology, University of California at Los Angeles, Los Angeles, California 90095, USA; 3 Department of Shanghai Institute of Cardiovascular Diseases, Zhongshan Hospital, Fudan University, Shanghai 200032, China; 4 Department of Bioinformatics, University of California at Los Angeles, Los Angeles, California 90095, USA; 5 Department of Sage Bionetworks, Seattle, Washignton 98109, USA; 6 Department of Medicine,University of California at Los Angeles, Los Angeles, California 90095, USA

**Keywords:** Proteome, Proteomics, Experimental models of disease, Mass spectrometry

## Abstract

Protein stability is a major regulatory principle of protein function and cellular homeostasis. Despite limited understanding on mechanisms, disruption of protein turnover is widely implicated in diverse pathologies from heart failure to neurodegenerations. Information on global protein dynamics therefore has the potential to expand the depth and scope of disease phenotyping and therapeutic strategies. Using an integrated platform of metabolic labeling, high-resolution mass spectrometry and computational analysis, we report here a comprehensive dataset of the *in vivo* half-life of 3,228 and the expression of 8,064 cardiac proteins, quantified under healthy and hypertrophic conditions across six mouse genetic strains commonly employed in biomedical research. We anticipate these data will aid in understanding key mitochondrial and metabolic pathways in heart diseases, and further serve as a reference for methodology development in dynamics studies in multiple organ systems.

## Background & Summary

Cellular proteomes are under constant insults. Regulation of proteome integrity requires chaperone-assisted folding of unfolded proteins, dissolution of misfolded aggregates, proteolytic removal of proteins, and other concerted proteostatic processes^1,2^. Recent studies have associated proteostatic disruptions causatively to an expanding list of disorders, including cystic fibrosis, neurodegenerations, and cardiovascular diseases^3–8^. In the heart, decreased proteolytic capacity and accumulating proteotoxcity have been shown to directly exacerbate outcomes in cardiac infarcts, hypertrophy, and failure^9,10^. Because proteostatic events often trigger zero net change in protein abundance but instead alter protein temporal dynamics^11–13^, they typically elude conventional experiments that measure only the steady-state abundance of proteins. Protein dynamics data are therefore sought to better describe homeostatic processes and enhance the utility of phenotyping-by-omics approaches. However, large-scale protein dynamics datasets have remained scarce, due to the specialized technologies necessary to measure turnover of individual proteins on a global scale.

We report here a large dataset of protein turnover dynamics in the heart of six common genetic strains of mice, acquired under both normal and hypertrophic conditions. The dataset contains over 1.92 million data points in protein isotope labeling kinetics, culminating as the *in vivo* turnover rates of 3,228 cardiac proteins and the expression levels of 8,064 proteins. Proteins with quantified dynamics belong to over 10 major cellular compartments and over 200 known pathways. Key proteins in mitochondria and metabolic pathways are encompassed, in addition to contractile machineries and sarcolemmal signaling proteins. To promote data reusability, we describe four example use cases where this dataset may be re-analyzed to support basic research, translational investigation, omics data integration, and kinetic modeling.

The present dataset was collected using a technology platform we recently developed, which overcame several technical challenges in quantifying individual protein turnover rates on a proteome scale. The history of protein dynamics traces back to 1935, when Schoenheimer and Rittenberg synthesized the first isotopologs of biological molecules to demonstrate the continuous renewal of proteins throughout life^14,15^. With recent progresses in shotgun proteomics, methodologies began to reach the sophistication and throughput required to understand turnover dynamics on a proteome scale. In contrast to steady-state protein abundance, which may be quantified directly in mass spectrometry (MS) by spectral intensity^16,17^ or sampling frequency^18,19^, protein turnover rates cannot be predicted from steady-state data^20^, requiring instead methods that can distinguish old and new protein molecules in mass spectra^11,21^. A common strategy is to introduce synthesized, isotope-tagged amino acids into cultured cells, such as in dynamic stable isotope labeling by amino acids in cell culture (dynamic SILAC) experiments that measure the time lapse required to fully label cellular proteins in culture^22,23^. Despite successful applications of dynamic SILAC *in vitro* in bacterial^24,25^, yeast^26^, and cultured mammalian cells^20,27–29^, protein turnover in freely-growing cultured cells does not recapitulate protein turnover in animals *in vivo*^8,30^. Intact animal studies with synthetic essential amino acids such as [^2^H_8_]-valine or [^13^C_6_]-lysine have been demonstrated to allow *in vivo* dynamics measurements^11,28^. These approaches have the advantage of labeling relatively immediate protein precursors, but require dietary modifications and may be financially costly in large studies. As an alternative strategy, we and others have proposed the use of minimal levels of deuterium oxide (^2^H_2_O) introduced in the drinking water of living animals to label their entire proteomes^12,13,31^.

In ^2^H_2_O labeling, protein turnover is measured as the rate of deuterium atom incorporation into the tissue protein pool following protein synthesis and degradation. The incorporation of deuterium is reflected as a gradual shift in peptide isotope clusters towards higher masses in mass spectra during the course of labeling^32–34^. We previously demonstrated several operational desiderata of ^2^H_2_O labeling, which include its safety, bio-orthogonality, ease of monitoring label enrichment, rapid clearance following label withdrawal^35,36^, and low cost in prolonged longitudinal studies^12,36,37^. We developed a data science software application, ProTurn, to solve the precursor-product relationship of peptide ions in mass spectra^38^ and automate large-scale ^2^H_2_O-labeled data analysis^12,37^. ProTurn reads in mass spectra and protein identification results to integrate the areas of all peptide isotope signals. Isotope patterns from multiple experimental time points are then tabulated for curve-fitting using a unified kinetic model, which corrects for potential labeling delays and computes turnover rates (*k*). The platform has proven applicable to recent mouse^12,37^ and human^36^ studies. The present study greatly expands on these previous efforts, encompassing the proteome-wide expression and dynamics of cardiac proteins across multiple sub-cellular compartments and six genetic backgrounds. All raw MS data have been deposited onto ProteomeXchange/PRIDE (PXD002870). Processed data tables and codes are available on Synapse (syn2289125), an open source platform for collaborative analyses provided by Sage Bionetworks.

## Methods

### Summary

**Figure 1:**
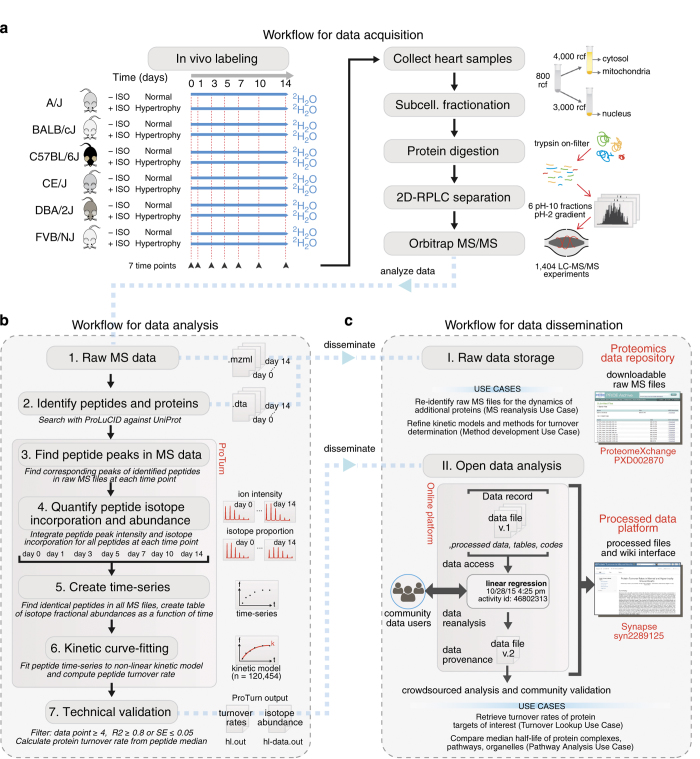
Workflows for data acquisition, analysis, and dissemination. (**a**) Flowchart of data acquisition, from *in vivo* labeling to the acquisition of Orbitrap mass spectrometry (MS) data. Normal and hypertrophic animals from A/J, BALB/cJ, C57BL/6J, CE/J, DBA/2J, and FVB/NJ mice were labeled for up to 14 days with ^2^H_2_O. A total of 78 sample groups were analyzed independently at 7 time points. From each group, hearts were excised and subjected to subcellular fractionation. Proteins were extracted for trypsin digestion and analyzed by high-resolution Orbitrap MS to measure isotope incorporation. (**b**) Flowchart of data analysis strategy, from MS data to technical validation. Raw mass spectra and protein identification results were analyzed by ProTurn. The intensity and isotope profiles of peptide mass spectra were quantified by integration over chromatographic spaces, then tabulated into time series and fitted to kinetic curves to deduce turnover rates, followed by stringency filters. (**c**) Data dissemination strategy, encompassing raw data storage and collaborative analysis platform. I. Raw data are deposited to a raw data repository for proteomics data, ProteomeXchange, where data users can download stored MS files to support raw data re-analysis and method development (see use cases 3 and 4 in text). II. The processed data and turnover rate tables are disseminated on an open data analysis platform, Synapse, where users can look up protein turnover rates and compare cellular pathways (see use cases 1 and 2 in text).

The overall strategy for data acquisition, analysis, and dissemination is summarized in Fig. 1.

### Animal models and *in vivo* isotope labeling

We performed ^2^H_2_O labeling and the cardiac hypertrophy model on A/J, BALB/cJ, C57BL/6J, CE/J, DBA/2J, and FVB/NJ mice. The animals (male, 9–12 weeks of age) were purchased from The Jackson Laboratory, and upon arrival were acclimatized at the UCLA housing facilities for 48 h. To initiate deuterium labeling, each animal received two intraperitoneal injections of 500-*μ*l 99.9% (molar ratio) ^2^H_2_O-saline spaced 4 h apart, at 12 noon and at 4 pm on the starting day of labeling, respectively (Fig. 1a). Following the injections, the animals had access *ad libitum* to 8% (v/v; 7.25% molar ratio) ^2^H_2_O in the drinking water supply for up to 14 days, along with standard lab chow (Harlan Teklad 7013). From each experimental group we euthanized two mice at each of day 0, 1, 3, 5, 7, 10, and 14 following the first ^2^H_2_O injection at 12:00 noon to collect heart and plasma samples. In the cardiac hypertrophy groups, we surgically implanted subcutaneous micro-osmotic pumps (Alzet) at the initiation of the first priming dose of ^2^H_2_O labeling^12^. The micro-osmotic pumps were calibrated to deliver 15 mg⋅kg^−1^⋅d^−1^ of isoproterenol over 14 days. All animal procedures were performed in accordance with the Guide for the Care and Use of Laboratory Animals by the National Research Council and approved by the Animal Research Committee at UCLA.

### Gas chromatography-mass spectrometry

To measure the rate and level of label enrichment in the animals, we performed gas chromatography-mass spectrometry (GC-MS) on the body water samples of the labeled animals. Mouse plasma (20 *μ*l) was mixed with 2 *μ*l of 10 N NaOH and 4 *μ*l of 5% (v/v) acetone in acetonitrile. In parallel, standard curves were created by adding, to the 5% acetone in acetonitrile, 0 to 20% (molar ratio) of ^2^H_2_O at 11 regular intervals in 1×phosphate-buffered saline in lieu of the mouse plasma. The sample and standard curve mixtures were incubated at room temperature for 12 h, after which the acetone portion was extracted by the introduction of 500 *μ*l of chloroform and 0.5 g of anhydrous sodium sulfate. The extracted solution (1 *μ*l) was analyzed directly by GC-MS (Agilent 6890/5975) using a J&W DB17-MS capillary column (Agilent, 30 m×0.25 mm×0.25 *μ*m). The column temperature gradient was as follows: 60 °C initial; then 20 °C⋅min^−1^ ramp to 100 °C; then 50 °C⋅min^−1^ ramp to 220 °C; then 1 min hold. The MS operated in the electron impact mode (70 eV) and selective ion monitoring at m/z 58 and 59 with 10 ms dwell time.

### Protein extraction and processing

Cardiac proteins were extracted following subcellular fractionation by differential centrifugation over density gradients^39^. Excised cardiac tissues were homogenized in an extraction buffer composed of 250 mM sucrose, 10 mM HEPES, 10 mM Tris, 1 mM EGTA, 10 mM dithiothreitol, and a protease/phosphatase inhibitor cocktail (Pierce Halt), at pH 7.4. The homogenate was centrifuged (800×g, 4 °C, 7 min). The pellet was collected and resuspended on 880 mM sucrose, 500 *μ*M MgCl_2_ at 3,000×g at 4 °C for 15 min and collected as the nuclear and extracellular fraction. The supernatant from the first (800×g) centrifugation step was again centrifuged (4,000×g, 4 °C, 30 min) to collect the supernatant as the organelle-depleted intracellular fraction. The pellet from this step was washed and centrifuged again (4,000×g, 4 °C, 30 min) to collect the pellets as the mitochondrial and microsomal fraction. We estimated protein yields using bicinchoninic acid assays (Thermo Pierce), then solubilized the protein fractions with RIPA buffer and performed proteolysis on 100 *μ*g protein from each fraction on 10,000-Da polyethersulfone filters (Nanosep; Pall Life Sciences). The RIPA buffer was exchanged on-filter with ammonium bicarbonate (100 mM, 100 *μ*l). Specifically, the samples were reduced (70 °C, 5 min) with dithiothreitol (3 mM) and alkylated in the dark (ambient temperature, 30 min) with iodoacetamide (9 mM). Proteins were digested on-filter (16 h, 37 °C) with sequencing-grade modified trypsin (50:1, Promega). Proteolysis was terminated and peptides were eluted by incubation with 20 *μ*l of 10% trifluoroacetic acid (Pierce) (30 min, 37 °C) followed by centrifugation (13,000×g, ambient temperature, 15 min).

### Liquid chromatography-tandem mass spectrometry

We analyzed the peptide samples by liquid chromatography-tandem mass spectrometry (LC-MS/MS) to discern peptide abundance, isotope incorporation, and sequences. To reduce sample complexity and increase protein coverage, we performed high-pH/low–pH two-dimensional reversed-phase chromatography to separate peptide samples prior to MS/MS^40,41^. The different pH values alter peptide charges to achieve orthogonal separation over hydrophobic stationary phases. First-dimension (high-pH) separation was conducted off-line on a Phenomenex C18 column (Jupiter Proteo C_12_, 4-*μ*m particle, 90-Å pore, 100 mm×1 mm dimension) at high pH using a Finnigan Surveyor liquid chromatography system. The solvent gradient profile was established by mixing solvent A (20 mM ammonium formate, pH 10) and solvent B (20 mM ammonium formate, 90% acetonitrile, pH 10) as follows: 0–2 min, 0–5% solvent B in solvent A; 3–32 min, 5–35% solvent B in solvent A; 32–37 min, 80% solvent B in solvent A; at 50 *μ*l⋅min^−1^ flow-rate. Fifty *μ*g of proteolytic peptides were injected with a syringe into a manual 6-port/2-position switch valve. Twelve fractions were collected every 2 min from min 16–40, then desiccated in a vacuum concentrator and re-dissolved in 20 *μ*l 0.5% formic acid with 2% acetonitrile prior to low-pH reversed-phase separation.

We performed on-line second-dimension (low-pH) reversed-phase chromatography on all samples using a single Easy-nLC 1000 nano-UPLC system (Thermo Scientific) on an EasySpray C18 column (PepMap, 3-*μ*m particle, 100-Å pore; 75 *μ*m×150 mm dimension; Thermo Scientific). Throughout the LC-MS/MS experiment, column temperature was held at a constant 50 °C. Each high-pH fraction was injected (10 *μ*l) and analyzed sequentially using the auto-sampler installed on the nano-UPLC system. The solvent gradient profile was established by mixing solvent A (0.1% formic acid, 2% acetonitrile) and solvent B (0.1% formic acid, 80% acetonitrile) as follows: 0–110 min: 0–40% solvent B in solvent A; 110–117 min: 40–80% solvent B in solvent A; 117–120 min: 80% solvent B in solvent A; at 300 nl⋅min^−1^. Column pressure was monitored to be within approximately 150 bar. High-resolution tandem mass spectrometry (MS/MS) was performed on a single LTQ Orbitrap Elite instrument (Thermo Scientific), coupled on-line to the nano-UPLC system through a Thermo EasySpray interface. MS signals were acquired in Fourier-Transform/Ion-Trap (FT/IT) mode: each FT MS1 survey scan was analyzed at 60,000 resolving power in profile mode, followed by rapid IT MS2 scans on the top 15 ions with monoisotopic peak selection. MS1 and MS2 target ion accumulation targets were 10^4^ and 10^6^, respectively. MS1 lock mass (m/z 425.120025) and dynamic exclusion (90 s) were used.

### Peptide identification and protein inference workflow

The acquired mass spectra were analyzed according to Fig. 1b. MS2 spectra were converted to.ms2 format using the MSConvert application from the ProteoWizard software package (v.2.1)^42^. Peptide identification was performed using the database search algorithm ProLuCID^43^ against a reverse-decoyed protein sequence database (Uniprot Reference Proteome *Mus Musculus*, reviewed, accessed April-08–2014, 16,672 forward entries and 16,672 decoy entries)^44^. Static cysteine carbamidomethylation (C +57.02146 Da) modification and up to three of the following variable modifications were allowed: methionine oxidation (M +15.9949 Da), lysine acetylation (K +42.0106 Da), serine/threonine/tyrosine phosphorylation (S/T/Y +79.9663 Da), lysine ubiquitylation (K +114.0403 Da), and asparagine deamidation (N +0.9840 Da). Tryptic, semi-tryptic, and non-tryptic peptides within a 20-ppm parent mass window surrounding the candidate precursor mass were searched. Peptide ions from up to 3 isotopic peaks with fragment mass tolerance of 600 ppm were allowed. Protein inference was performed by DTASelect v.2.0 (ref. 45), requiring ≤1% global peptide false discovery rate and 2 unique peptides per protein for the protein to be considered identified. Modified or non-tryptic peptides were subjected to separate statistical filters to limit false discovery using the –modstat and –trypstat options in DTASelect, such that the inclusion of the variable modifications had no negative impact on the total protein identification counts.

### Kinetic data processing strategy

We analyzed protein turnover kinetics and estimated fitting errors using a method we previously described^12,37^. ProTurn automatically retrieved identified peptides that were uniquely assigned to proteins for area integration. Specifically, the acquired Orbitrap mass spectra in Thermo .raw formats were first converted into. mzML format using ProteoWizard (v.2.1)^42^, then input to ProTurn (v.2.0.5) for analysis. The ProTurn parameters were set as follows: area-under-curve integration width: 60 p.p.m., extracted ion chromatograph smoothing: Savitzky-Golay filter^46^ over 7 data points. To further control against peptide false positive identifications, only peptides that were explicitly identified (1% FDR) and integrated in ≥4 time points were accepted for the calculation of protein abundance and turnover. The ‘Allow Peptide Modification’ option in ProTurn was turned on to include any potentially identified post-translationally modified (PTM) peptides in kinetic curve-fitting.

We fitted the kinetic data using the non-steady-state fitting method in ProTurn, which corrects for any time delay in label enrichment in the experimental model using a first-order kinetic curve to approximate the equilibration of ^2^H_2_O in the total body water. The enrichment curve is described by two parameters: the rate (*k*_*p*_) and plateau level (*p*_*ss*_) of deuterium enrichment in body water, both of which were empirically derived from GC-MS measurements at the sampled time points as described above, such that:

(1)p=pss⋅(1−e−kpt)

The GC-MS data on enrichment kinetics were utilized in the unified kinetic equation to calculate the corrected fraction of newly synthesized peptides from the isotope envelope fractional abundance, which accounts for the fewer labels contributed by a newly synthesized protein early in the labeling procedure. This correction does not shift the kinetic curve rightward but instead retards its initial ascent to the plateau. Briefly, the overall change in the fraction of unlabeled peptide isotopomers in a peptide pool is assumed to follow first-order kinetics:

(2)dA0dt=k⋅(A0,max−A0)

where *A*_0_ is the fraction of the 0th isotopomer of a peptide isotope envelope at a given time, and *A*_0,*max*_ is the fraction of the 0th isotopomer of a pool of newly synthesized peptides. The value for *A*_0,*max*_ is given by *A*_0,*max*_=*a*⋅(1–*p*)^*N*^ where *a* is the fraction of the 0th isotopomer in unlabeled samples as calculated by the natural abundance of heavy isotopes^47^ and *N* is the number of labeling sites in the peptide sequences as estimated according to literature values^48^. Further substituting into equation (1) and equation (2) gives a combined equation with two kinetic rate constants:

(3)dA0dt=k⋅(a(1−pss⋅(1−e−kpt))N−A0)

The integrated rate equation for equation (3) can be derived analytically as described in our previous publication^12^, and is used in ProTurn optimizations to derive the turnover rate constants (*k*) of peptides (see below). The GC-MS measurements of animal labeling kinetics used to calculate *p*_*ss*_ and *k*_*p*_ are deposited on Synapse (ID: syn4847184).

To calculate peptide isotope cluster abundance in ProTurn, the summed area of the peptide isotope envelope was normalized to total spectral intensity, then normalized to the number of possible tryptic peptides 6–30 amino acids in length from *in silico* digestion of the protein^17^, and further normalized to the total relative areas within the sample. To calculate peptide turnover rates, the fractional abundance of the 0th mass isotopomer (m_0_) from each integrated time point was modeled with the combined kinetic model above. To estimate the values of *k*, the model was iteratively fitted to the data points to minimize the squared residual values using the Nelder-Mead simplex method^49^. Gradient optimization using the Broyden-Fletcher-Goldfarb-Shanno method^50^ yielded same optimized values of *k* in our kinetic model. Peptide isotopomer time-series with *R*^2^≥0.8 or standard error of estimate (s.e.) ≤0.05 are considered to pass the stringency filter employed here, but all peptide time-series data are made available regardless of their *R*^2^ or s.e. values. Protein turnover rates are reported as the median and median absolute deviation of the optimized turnover rate constants of all accepted constituent peptides.

### Code availability

All software packages used for processing MS data are publicly available. ProTurn (v.2.0.5) is freely available on our website (http://heartproteome.org/proturn). Additional data analysis codes that perform summary statistics and create the presented figures were written in R (v.3.2.1) and may be freely accessed on Synapse (ID: syn2289125).

## Data Records

### Quantification of absolute *in vivo* cardiac protein turnover rates from mass spectrometry data

We acquired the dataset with the goal of examining the temporal dynamics of the mammalian heart proteome. Because protein expression is known to be highly variable among naturally occurring healthy genetic backgrounds of the same species^51–53^, the genetic composition of animals on which experimentation is performed can impact the generalizability of discoveries from one experimental model to another. We therefore replicated the proteomics analysis of cardiac tissue samples six times in total (A/J, BALB/cJ, C57BL/6J, CE/J, DBA/2J, FVB/NJ). The six strains were chosen for their common usage in diverse genetic, surgical, and pharmacological models in human disease research, with noted prevalence of A/J in immunological studies, BALB/cJ mice in cancer and cardiovascular research; CE/J mice in cancer, neurobiological, and metabolic research, DBA/2J mice in cardiovascular and developmental studies; and C57BL/6J and FVB/NJ mice in general-purpose and genetics applications^54^.

The entire dataset comprises 1,404 LC-MS/MS experiments performed over eight months of instrument time. All raw MS data files (in Thermo .raw files format) used for protein identification and quantification have been deposited to the PRIDE repository in the ProteomeXchange Consortium^55^ and can be accessed with the dataset identifier PXD002870 (Table 1) (Fig. 1c) (Data Citation 1). A compressed archive is also available on PRIDE that contains all protein identification results from ProLuCID/DTASelect. All protein database search results including peptide and protein identification are recorded on Synapse^56^, with the dataset identifier syn2289125 (Data Citation 2).

**Figure 2:**
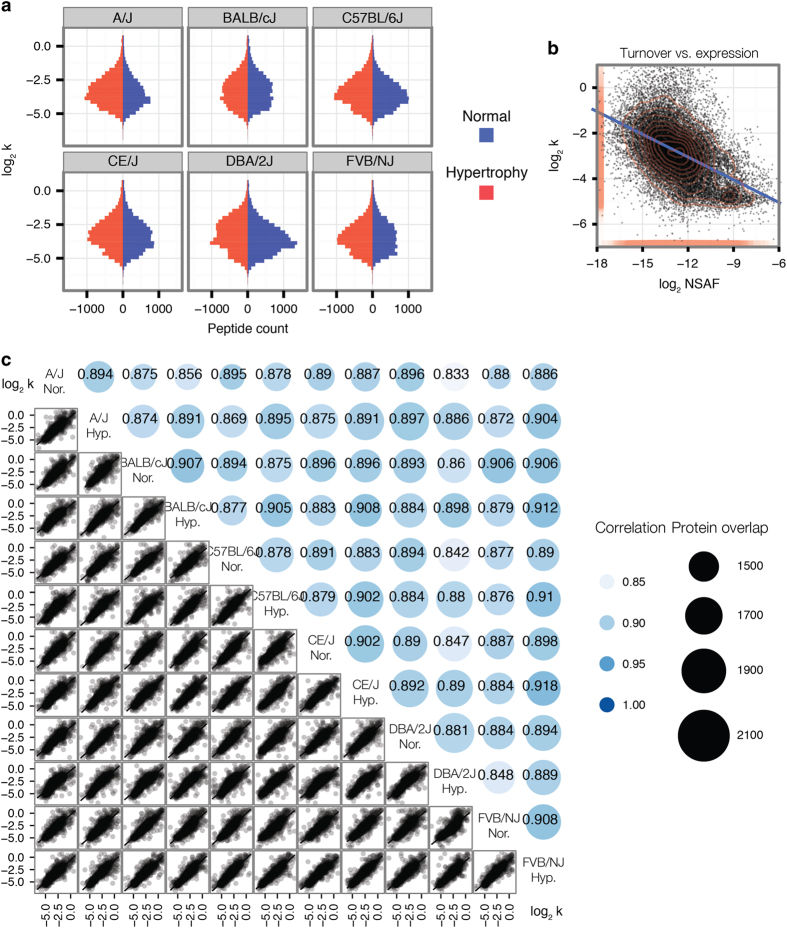
Distributions of measured protein expression and turnover rates. (**a**) Histograms of turnover rates in log_2_ space. Histograms show the distribution of turnover rates in each of the six mouse genetic strains under both normal and hypertrophy conditions. As each peptide time-series is fitted independently, the turnover rates of individual peptide time-series are presented in this figure for clarity. Subsequently, protein turnover rates are calculated as the median of the turnover rates of all constituent peptides. Blue histograms: normal hearts. Red histograms: hypertrophy hearts. Horizontal axis: peptide counts. Vertical axis: turnover rate (k) (d^−1^). (**b**) Correlations between protein turnover rates and protein abundance. Protein turnover rates are calculated as the median of turnover rates of all constituent peptides. The scatter plot between the log_2_ abundance (x) versus log_2_ turnover rate (y) of proteins shows an expected negative correlation between protein turnover and abundance. Rug and contour: data density. Line linear regression. (**c**) Correlation matrix of turnover rates in normal and hypertrophy hearts of six mouse strains. The lower triangle of the matrix contains pair-wise scatter plots of log_2_ turnover rates of shared proteins between two samples. The upper triangle of the matrix shows numbers of shared proteins (sizes) and Spearman’s correlation coefficients of each pairwise comparison (colors and figures).

In total, we acquired over 1.92 million data points of quantified peptide isotopomer distributions corresponding to 341,353 peptide time series quantified at 4 or more time points over the course of labeling. We derived confident kinetic rate constants from 120,454 peptide time-series that passed our stringency filter (see ‘Technical Validation’ for discussions on filter selection below) (Data Citation 3). Label enrichment kinetics from GC-MS data, used to calculate peptide turnover rates, are available on Synapse (Data Citation 4). The peptides have median R^2^ of 0.93 (interdecile range: 0.84–0.99) and median standard errors of 7.1% (interdecile range: 4.3–11.9%). Protein turnover rates were calculated as the median of the turnover rates of all member peptides that passed our stringency filter. The turnover kinetics of 3,228 cardiac proteins were quantified at a total of 23,929 protein-sample pairs (Table 2), averaging 7.4 samples in which each protein was quantified, with 2,599 (81%) proteins that were quantified in at least three samples, and 863 (27%) proteins quantified in all 12 samples. The median turnover rate of the quantified peptides is 0.094 d^−1^ (9.4% replacement, half-life 7.3 d) with interdecile range of 0.037 d^−1^ to 0.30 d^−1^ (half-life 2.3 d to 18.9 d) (Fig. 2a).

The dataset includes protein dynamics information from diverse cellular compartments and 201 non-overlapping known cellular pathway groups (Tables 3 and  4). We retrieved pathway information from Reactome (release V53; July 2nd, 2015)^57^. Pathways are considered to be covered in the dataset if five or more proteins in the pathway contain quantified half-life information amongst the 3,228 proteins with quantified half-life. Pathways with 50% or more overlap are combined into pathway groups. The dataset captures turnover rates that span >20-fold, and includes long-lived proteins such as histone H4 (Uniprot ID P62806; median half-life 54.6 days) and lamin-B1 (Uniprot ID P14733; median half-life 36.4 days), as well as fast turnover proteins such as apolipoprotein E (Uniprot ID P08226; median half-life 8 h). As previously reported, abundant proteins in the dataset have slower turnover on average (Fig. 2b). Data from each of the six mouse strains under normal and hypertrophy conditions show good correlations and overlaps (Fig. 2c).

The turnover rate output files from ProTurn have been uploaded to Synapse for public access and collaborative data analysis (syn2289125), including data tables on the outputs of all protein turnover calculations. An index file linking each sample to individual data table can be found at ID: syn4725236. Each ProTurn analysis contains three output files, which contains protein half-life information organized by peptide sequence (hl.out); isotopomer envelope quantities at each time point (hl-data.out); and the total normalized intensity of each protein in each MS experiment for protein quantification purposes (intensity.txt). The hl.out files contain 14 columns; each row is a unique peptide (sequence- and charge- unique) time series that passed the time point filter. The column labels are as follows: ID: internal index for referring to corresponding data in hl.out. UniProt: UniProt ID of the protein the peptide was assigned to in database search. Peptide: sequence (with PTM, if applicable). DP: number of data points. This corresponds to the number of time points in most runs. z: peptide ion charge state. mi: index of the isotopomer whose proportional abundance is quantified (0 in most ProTurn analyses). SS: residual sum of squares of fitting. a: initial isotopomer fractional abundance prior to labeling based on peptide sequence. pss: experimental steady-state relative label enrichment level. kp: experimental rate constant of label enrichment (d^−1^). N: number of accessible labeling sites based on sequence information, as calculated according to literature values^48^. k: fitted rate constant of peptide turnover (d^−1^). dk: fitting error. R2: goodness-of-fit. The hl-data.out files contain 3 columns; each row is a unique peptide isotope cluster at a particular time point. The columns are as follow: ID: internal ID for referring to corresponding data in hl.out; t: time point (d); A_0_: experimental fractional abundance of the isotopomer. The intensity.txt files contain eight columns, with the first column being the Uniprot ID of the protein, and each successive column thereafter denoting the relative intensity of the protein in each of the examined time point of analysis respectively (day 0, day 1, day 3, day 5, day 7, day 10, day 14).

### Quantification of relative cardiac protein expression from mass spectrometry data

The dataset also provides protein expression information from two parallel methods of label-free quantification, based on the extracted ion chromatogram (XIC) intensity-based quantification carried out natively in ProTurn, and the normalized spectral abundance factor (NSAF) from the proteomics database search workflow ProLuCID/DTASelect^43,45^. The XIC intensity-based quantification is normalized by the number of tryptic peptides that a protein is estimated to produce^17^. From the 8,227 identified proteins, ProTurn quantified the expression levels of 8,064 proteins by XIC areas-under-curves in over 58,748 protein-samples, averaging 7.29 samples in which each protein was quantified. A total of 5,806 (72%) proteins were quantified in at least three samples and 3,205 (40%) were quantified in all 12 samples. In parallel, the spectral count methods quantified the abundance of 3,484 proteins in at least three samples. Results from the two methods are correlated (Spearman’s correlation coefficient *ρ*: 0.73), but XIC intensity-based quantification achieves a broader dynamic range than spectral counts.

An index file containing all individual protein identification results and expression quantification values is uploaded at Synapse (syn4720849). Each record is in standard text format as output from ProLuCID/DTASelect without modification. The files contain search metadata, and, in each row, a peptide or protein identification record containing scores, spectral counts, mass shift, and other properties as described.

## Technical Validation

### Strategies for data quality assurance

**Figure 3:**
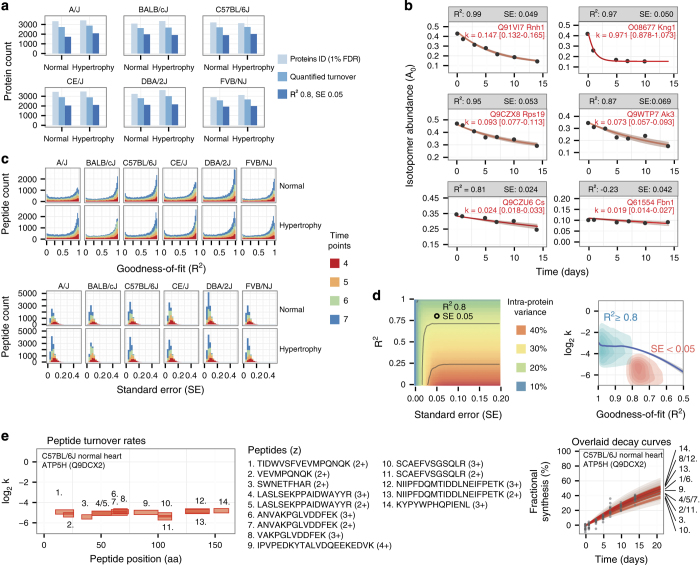
Technical Validation of acquired turnover rates. (**a**) Bar charts showing in each of the samples: the number of proteins (i) identified at 1% FDR (light blue), (ii) quantified with isotope incorporation data at ≥4 time points (blue), and (iii) quantified with derived turnover rates passing stringency filters (dark blue). (**b**) Peptide decay curves across a range of goodness-of-fit (R^2^) and standard error (s.e.) values. Panels show experimental data of the fractional abundance of the 0th peptide isotopomers (A_0_) (y) over time (x), illustrating representative qualities of fitting at various R^2^ and s.e. values passing the stringency filter. Red line: best-fit kinetic curve. Red area: upper and lower bounds of fitting. (**c**) Histograms of R^2^ (top) and s.e. (bottom) for the fitted peptide data. The R^2^ histograms include all quantified peptides; s.e. histograms include only peptides passing stringency filters. Colors of stacked histograms reflect the number of time points at which the peptide’s isotope fractional abundance was quantified. (**d**) (Left panel) Cut-offs at various values of s.e. (x) and R^2^ (y) were sampled stepwise to determine their effects on the intra-protein variance of turnover rates (heatmap colors), calculated as the median of the median absolute deviations of turnover rates of peptides identified to the same proteins. Using R^2^ as sole filter excludes a subset of well-fitted peptides (lower left). (Right panel) Density plot showing distribution of R^2^ (x) versus log_2_ turnover rate (y) for peptides passing the stringency filter. Colors of density contours denote two groups of accepted peptides (blue: R^2^≥0.8; red: s.e. ≤0.05). Blue line: local regression. Accepted peptides with R^2^<0.8 have lower turnover rates. (**e**) Turnover rates of 14 distinct peptides from one protein (ATP5H). (Left) The amino acid (aa) position and length (x) of the peptides along the protein sequence are plotted against log_2_ turnover rates of the peptides (y), showing consistent turnover. Indices refer to peptide sequences in the middle. (Right) Overlaid decay curves for the 14 ATP5H peptides. Isotope abundance (A_0_) is rescaled to fractional synthesis (y) to normalize the position of each peptide curve over time (x).

To assure data quality, we use a multi-step strategy (see Methods), selecting only proteins that are identified at 1% FDR and quantified at 4 or more data points for curve-fitting, and accepting only the turnover rate values that pass a stringency filter (Fig. 3a). Data accuracy may be assessed both with regard to mass spectrometry measurements (i.e., whether the mass spectrometer measures the correct isotopomer fractional abundance for a given peptide) and to biological reality (e.g., whether the measured turnover rate accurately reflects the true turnover rate *in vivo*). To determine the former, we investigated the accuracy of experimental measurements of peptide isotopomer relative abundance in unlabeled sample against theoretical isotope distributions, which can be calculated from peptide sequences and the natural abundance of heavy isotopes in the biosphere^47^. We find good agreement between theoretical and experimental values, with Spearman’s correlation coefficient *ρ* of 0.97 and root mean square error between experimental and theoretical isotope abundance of 2.6%, supporting overall excellent accuracy in the measurement of isotope relative abundance on mass spectra.

Because gold standards of *in vivo* protein turnover rates are largely unavailable, we assayed the biological validity of the dataset using indirect methods. First, we derived and optimized data filtering criteria to maximize the number of peptides and proteins with quantitative information while simultaneously controlling intra-protein variability. These filter targets are predicated on the assumptions that for most scenarios the entire protein sequence are synthesized and degraded as a unit, and that turnover measurements are self-normalized and insensitive to differential yields across experimental fractions. Hence, multiple quantified peptides originating from the same measured cellular protein pool ought to yield identical turnover rates if data quality is pristine, barring any unresolved isoforms or identification ambiguity. We used goodness-of-fit (R^2^) as the primary criterion to filter out the peptide time-series in which the experimental A_0_ data points deviated significantly from the optimized kinetic curves (Fig. 3b). Relaxing the acceptable R^2^ value to below 0.8 resulted in gradual admittance of peptide time-series with increasing residuals, which in turn led to increases in intra-protein variances. In our experience, we found intra-protein variance of <30% in an experiment to be acceptable, when measured as the median of the median absolute deviations of best-fitted peptide turnover rate constants within each protein in an experiment. Peptide time series that are quantified at more time points show better fitting quality (Fig. 3c). On average, 35% of peptides were fitted with R^2^≥0.8. In peptides that were quantified in more time points, a higher proportion was closely fitted to the curves (R^2^≥0.8) (31.2% of peptides quantified in 4 time points; 31.8% of peptides quantified in 5 time points; 35.1% of peptides quantified in 6 time points; 40.5% of peptides quantified in 7 time points). As expected, peptides passing the stringency filters which are quantified in more time points also show lower standard errors of estimate (s.e.) (Fig. 3c). This discrepancy may be due to peptides possessing higher mass spectral intensity leading to improved detection as well as more accurate peak area integration.

Peptides undergoing relatively slow turnover, such as those originating from long-lived nuclear and mitochondrial proteins, lend to flat and horizontal kinetic curves (Fig. 3b, lower right panel). In these peptides, the kinetic curve has limited power to predict the value of A_0_ within the studied timeframe, and consequently results in high residual variances. We therefore used a second filter based on total variances of the data points to include peptides with slower turnover and extend the overall dynamic range of the dataset. Stepwise permutation of R^2^ and s.e. values (Fig. 3d) suggest that a small subset of peptides with low R^2^ values are nevertheless well-fitted and contribute to consistent intra-protein turnover rates. These peptides are distinguished by their lower average turnover rates (Fig. 3d). To balance the quality and quantity of protein turnover measurements, we use a stringency filter of R^2^≥0.8 in the analysis here, and additionally include peptides whose standard errors are ≤0.05. This filter preserves a reasonable fraction of fitted peptides, whereas accepted peptides from each protein demonstrate consistent turnover rates, as can be seen in 14 distinct peptides independently measured from mitochondrial ATP synthase subunit d (ATP5H) (Fig. 3e). Other stringent filters may be easily applied to the raw data by the data consumers, if intra-protein variance is to be further minimized.

## Usage Notes

### Exploration of turnover kinetics in protein pathways

The mouse strain data documented here may be used to explore natural variations in protein expression and dynamics across healthy genetic backgrounds in a population, whereas the isoproterenol-treated samples may be used to explore variations in pathological responses towards a common stimulus. A number of post-analysis routes are available with the current dataset. For instance, the absolute turnover rate data may be used to interrogate whether particular protein pathways or sub-proteome (such as from an immunoprecipitation study) are co-regulated in their expression and turnover, which may be used to inform on regulatory mechanisms. Subcellular or extracellular localization may influence protein half-life due to the predominant proteolytic mechanisms presented in various compartments. For some proteins, exit from the tissue-of-origin may represent a considerable removal mechanism, which should be taken into account when interpreting data on proteins for which the total equilibrating pool may not reside entirely within the sampled proteome. Exported proteins may appear to have shorter half-life in the heart than when measured in extracellular locations (e.g., in blood), if it may be assumed that a majority of the proteins are quickly lost from the intracellular pool upon their synthesis in the heart. Under rapid export, only recently synthesized proteins may be sampled within the heart, and the sampled proteins resemble the completely turned-over protein pool (i.e., *A*_0,*t*_ resembles *A*_0,*max*_), a relationship which has been exploited to measure label enrichment of precursor pools from exported liver protein products^58^. The heart is not a major secretory organ, but a small number of proteins are actively secreted from the heart in health or in disease (e.g., natriuretic peptides A and B plus certain cytokines^59^) whereas other proteins may be passively shredded upon cell death or injury (e.g., cardiac troponin I and T^60^). Thus a number of different pathophysiological scenarios including protein differential expression, export, and loss may manifest as alterations in effective turnover rates. Kinetic information may also be used in computational modeling of cellular processes that require kinetic rate constants as input parameters, such as in the simulation of protein damage, protein homeostasis and long-term alterations in expression^61^, the stability of disease markers and agonist receptors^62^, or the stochasticity of transcript/protein expression regulation *in vivo*^25,29^.

### Alternative routes for data analysis

We present the following four specific examples of use cases in both basic and translational investigations as preliminary guidance on how the data may be analyzed or re-analyzed to gain biological insights. Specific step-by-step instructions of the four use cases below can be found on COPaKB (http://heartproteome.org) and on Synapse (http://doi.org/doi:10.7303/syn2289125).

Use case 1: Turnover Lookup. The half-life and abundance of proteins may be easily and individually retrieved via the data records on the Synapse project (http://doi.org/doi:10.7303/syn2289125) to support targeted queries. An investigator in cardiovascular medicine may be interested in mitochondrial biology in cardiovascular diseases. He or she may study a particular cardiac protein, such as mitofusin 2 (Uniprot ID: Q80U63), and its role in mitochondrial turnover during the development of heart failure. The absolute half-life of the protein in the heart can be looked up by downloading the protein turnover rate tables (e.g., see syn4725236 for an index of result files from all strains) and querying it with the protein’s UniProt ID for various investigation objectives, such as to calibrate the rate of decay of fluorescence timers in other experimental systems with the absolute time of mitochondrial turnover in the intact heart. It may also be possible to study the relationship between the half-life of mitofusin with that of the mitochondrial median and use the information to contextualize whole-mitochondrion dynamics data. Combined with additional data on the absolute quantification of mitofusin, one may calculate the absolute copy numbers of mitofusins that are being synthesized and degraded, and how they relate to mitochondrial turnover rates.

Use case 2: Pathway Analysis. Protein complexes, pathways, and cellular compartments may be analyzed to understand their individual protein half-life and expression. A basic scientist who studies Golgi proteins in the yeast may utilize the data records to ask fundamental questions on the cell biology of the organelle. For example, the median half-life of all Golgi proteins may be assayed and contrasted with other cellular components such as the endoplasmic reticulum (Table 3). Protein half-life and expression in relevant pathways may be retrieved (Table 4). Moreover, because biological fractionation was performed in acquiring this dataset, raw MS data files on PRIDE may be re-analyzed separately to compare the half-life of potential protein subpopulations that may be enriched in either the nuclear/insoluble, cytosol, or mitochondrial/ER isolations. In our previous investigations, we have noticed potential differences in hexokinase isoforms when assayed from cytosol and from the mitochondria^34^.

Use case 3: MS Re-analysis. Proteomics data form an important component of omics-based disease phenotyping strategies. Nevertheless, it is generally appreciated that substantial amounts of information remain unextracted in shotgun proteomics datasets, in the form of unidentified spectra not matched to any peptides in the utilized protein identification workflow. By our estimation, more than half of the MS2 spectra in the uploaded .raw files may be unidentified under the protein identification filters we used. An omics scientist or informatician interested in proteome dynamics may therefore re-analyze the uploaded raw MS data with more sophisticated protein identification workflows in the future. For instance, one may explore the kinetic regulations of single amino acid variants using proteogenomics databases^63^, or unknown protein identifications with improved search engine parameters^64^. The re-analyzed data may be further combined with available complementary omics data (e.g., microarray data) from identical models for deeper insights. To support omics data re-analysis, we have built into ProTurn compatibility with database search results from multiple common search engines including Mascot (Matrix Science), SEQUEST/ProLuCID^43,45^, MaxQuant/Andromeda^65^, ProteomeDiscoverer (Thermo), and COPaKB^66^.

Use case 4: Method development. This study provides a complete reference dataset from raw data to quantitative results, thus presenting opportunities for new software and kinetic models for protein turnover analysis to be developed and tested. Data analytical methods on the detection of differential protein turnover remain at infancy. Data scientists interested in protein dynamics may reanalyze the quantified peptide time-series for novel insights, for instance, on the behaviors of peptide series that do not conform to the current kinetic model or pass the current stringency filters. It is known that some proteins may have sequential, biphasic responses to stimuli^20,67^, which may cause deviation from our kinetic model. On the other hand, some long-lived proteins including histones and nucleopore channel subunits^8,68^ may not accumulate sufficient deuterium atoms during the labeling time period, and may be identified by data analysis routes that specifically target the absence of labels. We anticipate that further method developments will lead to a virtuous cycle of reusability of the proteome dynamics data in this dataset.

In summary, we describe here one of the largest experimental datasets on proteome dynamics in an animal model of human diseases, covering over 10 major organelles and 200 distinct cellular pathways. We envision the data will provide new molecular information on disease phenotypes and support further development in dynamics research.

## Additional Information

**How to cite this article:** Lau, E. *et al.* A large dataset of protein dynamics in the mammalian heart proteome. *Sci. Data* 3:160015 doi: 10.1038/sdata.2016.15 (2016).

## Supplementary Material



## Figures and Tables

**Table 1:** Samples and Experimental Files in the Dataset.

**Strain**	**Condition**	**Animals**	**DP**	**MS files**	**Raw data**	**Proc. data**
A/J	Normal	14	7	126	PXD002870	syn4509334
A/J	Hypertrophy	12	6	108	PXD002870	syn4591707
BALB/cJ	Normal	14	7	126	PXD002870	syn4591751
BALB/cJ	Hypertrophy	12	6	108	PXD002870	syn4591754
C57BL/6J	Normal	14	7	126	PXD002870	syn4591893
C57BL/6J	Hypertrophy	12	6	108	PXD002870	syn4591895
CE/J	Normal	14	7	126	PXD002870	syn4591887
CE/J	Hypertrophy	12	6	108	PXD002870	syn4591889
DBA/2J	Normal	14	7	126	PXD002870	syn4591737
DBA/2J	Hypertrophy	12	6	108	PXD002870	syn4591741
FVB/NJ	Normal	14	7	126	PXD002870	syn4591761
FVB/NJ	Hypertrophy	12	6	108	PXD002870	syn4591863
	Total	156	78	1,404		
DP Number of time points from which labeled samples were collected. Proc. Data: Processed Data. Additional details can be found in the metadata.csv file. MS files: number of mass spectrometry raw files. Raw data: raw data are individual data files deposited on ProteomeXchange/PRIDE under the ID PXD002870; processed data are deposited on Synapse.						

**Table 2:** Protein identification and quantification by sample in the dataset.

**Strain**	**Condition**	**Identified**	**Quantified**	**Filtered**
A/J	Normal	3,336	2,734	1,733
A/J	Hypertrophy	3,421	2,882	2,085
BALB/cJ	Normal	3,092	2,630	1,982
BALB/cJ	Hypertrophy	3,353	2,678	1,911
C57BL/6J	Normal	3,213	2,704	1,896
C57BL/6J	Hypertrophy	3,134	2,744	2,018
CE/J	Normal	3,452	2,898	2,037
CE/J	Hypertrophy	3,485	2,884	2,095
DBA/2J	Normal	3,245	2,799	2,109
DBA/2J	Hypertrophy	3,638	3,002	2,159
FVB/NJ	Normal	2,896	2,570	1,921
FVB/NJ	Hypertrophy	3,127	2,665	1,983
Identified: Average number of proteins identified at 1% FDR. Quantified: Number of proteins with quantified turnover. Filtered: Number of proteins with quantified turnover rates passing the employed stringency filter (R^2^≥0.8, s.e. ≤0.05) (see text).				

**Table 3:** Selected major organelles and cellular components covered in this dataset.

**GO ID**	**Name**	**# Proteins**	**Adjusted** ***P***
GO:0005739	Mitochondrion	524	1.7e−290
GO:0005829	Cytosol	375	9.2e−168
GO:0005634	Nucleus	588	9.5e−149
GO:0005886	Plasma Membrane	331	1.2e−77
GO:0005615	Extracellular Space	202	1.3e−71
GO:0005783	Endoplasmic Reticulum	147	1.9e−53
GO:0005794	Golgi Apparatus	114	9.5e−33
GO:0005768	Endosome	50	8.7e−21
GO:0005777	Peroxisome	35	1.3e−18
GO:0005764	Lysosome	45	4.4e−18
Adjusted *P*: Benjamini-Hochberg adjusted *P* value for hypergeometric tests for enrichment of the cellular compartment against the entire mouse proteome on Uniprot.			

**Table 4:** Selected biological pathways covered in this dataset.

Pathway Group*	**Representative Names**	**Proteins**
R-MMU-2467813 R-MMU-983168 R-MMU-195253 R-MMU-5632684 R-MMU-450408 R-MMU-5607764 ...(37)	Antigen processing: Ubiquitination & Proteasome degradation	132
R-MMU-72706 R-MMU-156827 R-MMU-1799339 R-MMU-72689 R-MMU-975957 R-MMU-975956 ...(11)	GTP hydrolysis and joining of the 60S ribosomal subunit, SRP-dependent cotranslational protein targeting to membrane	107
R-MMU-611105 R-MMU-611105	Respiratory electron transport	78
R-MMU-5389840 R-MMU-5419276 R-MMU-5368286 R-MMU-5389840	Mitochondrial translation elongation	69
R-MMU-72163 R-MMU-72163	mRNA Splicing major pathway	49
R-MMU-114608 R-MMU-114608	Platelet degranulation	45
R-MMU-5628897 R-MMU-5628897	TP53 Regulates Metabolic Genes	44
R-MMU-72695 R-MMU-72695	Formation of the ternary complex, and subsequently, the 43S complex	42
R-MMU-1445148 R-MMU-1445148	Translocation of GLUT4 to the plasma membrane	40
R-MMU-216083 R-MMU-3000178 R-MMU-216083	Integrin cell surface interactions	38
R-MMU-2132295 R-MMU-2132295	MHC class II antigen presentation	37
R-MMU-5663220 R-MMU-2500257 R-MMU-68877 R-MMU-5663220	RHO GTPases Activate Formins	37
R-MMU-1268020 R-MMU-1268020	Mitochondrial protein import	35
R-MMU-2565942 R-MMU-5620912 R-MMU-380270 R-MMU-380259 R-MMU-380284 R-MMU-2565942	Regulation of PLK1 Activity at G2/M Transition	31
R-MMU-3371453 R-MMU-3371453	Regulation of HSF1-mediated heat shock response	27
R-MMU-1650814 R-MMU-1442490 R-MMU-2022090 R-MMU-1650814	Collagen biosynthesis and modifying enzymes	27
R-MMU-70263 R-MMU-70263	Gluconeogenesis	23
R-MMU-5625740 R-MMU-5627123 R-MMU-5627117	RHO GTPases activate PKNs	23
R-MMU-432722 R-MMU-432722	Golgi Associated Vesicle Biogenesis	22
R-MMU-2029482 R-MMU-5663213 R-MMU-3928662 R-MMU-2029482	Regulation of actin dynamics for phagocytic cup formation	22
Pathway Groups: Reactome pathway accession numbers are members of groups of pathways that shared 50% or higher overlap in their associated proteins. Only pathways with five or more proteins in this dataset are included. Only the top 20 of the 201 pathway groups covered in the dataset are shown in this table.		
